# Supplementary material for: High-efficiency magnetic refrigeration using holmium
Source: Nat Commun. 2021 Feb 19;12:1212. doi: 10.1038/s41467-021-21234-z (PMC7896054; doi:10.1038/s41467-021-21234-z)
Supplement: Supplementary file 1 — Supplementary Information [file 41467_2021_21234_MOESM1_ESM.pdf]

High-Efficiency Magnetic Refrigeration Using Holmium

Noriki Terada and Hiroaki Mamiya

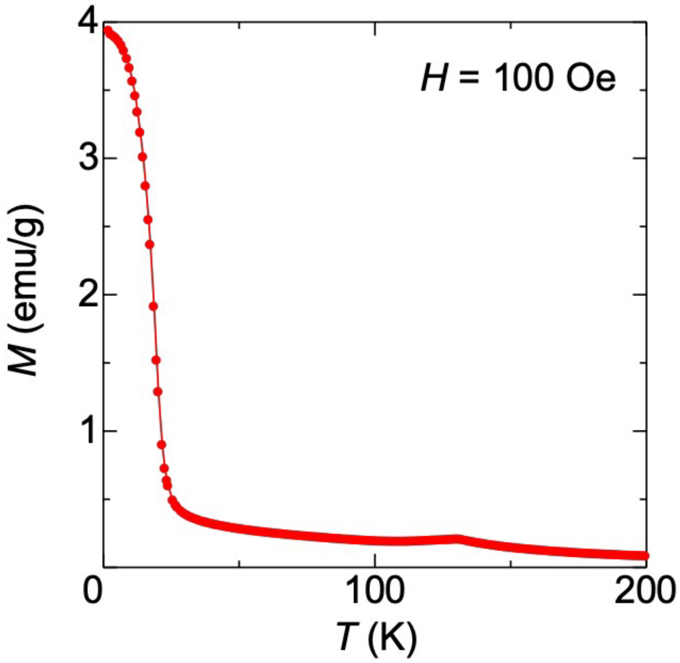

Supplementary Figure 1: Temperature dependence of magnetization in a magnetic field of 100 Oe in Ho.

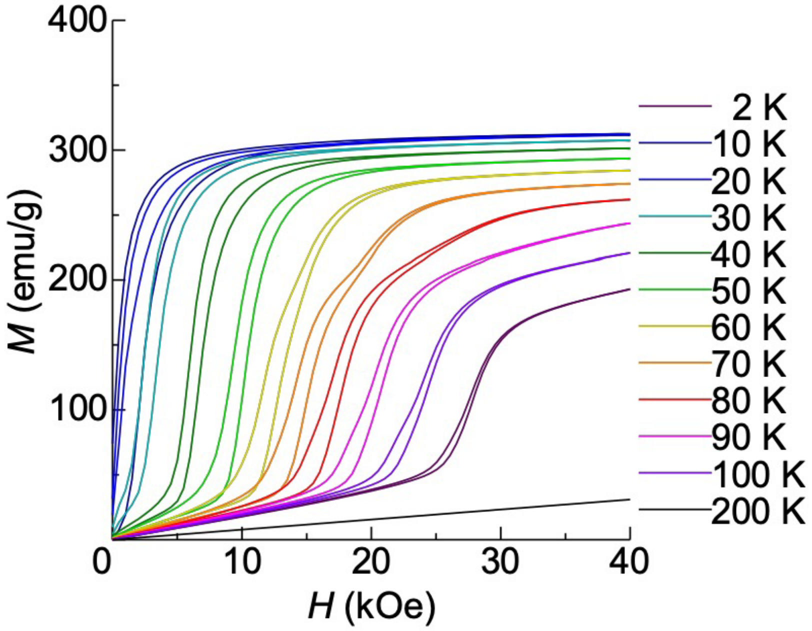

Supplementary Figure 2: Magnetization curve at typical temperature in Ho. These data are identical to those in Fig. 2 in the main text. The unit of magnetization is emu/g.

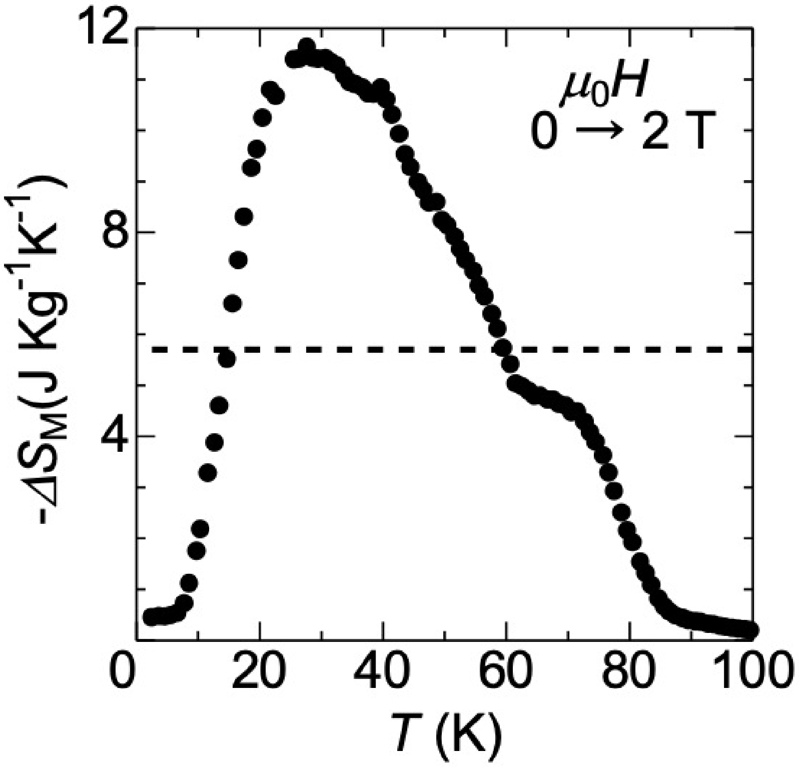

Supplementary Figure 3: Temperature dependence of negative magnetic entropy change for a magnetic field change from $\mu_0 H = 0$ T to $\mu_0 H = 2$ T in Ho. From this data, the relative cooling power (RCP), defined as $RCP = \Delta S_M^{\max} \cdot \delta T_{\rm FWHM}$, can be estimated to $RCP \sim 0.5$ kJ/kg in $\mu_0 H = 2$ T in Ho, where $\Delta S_M^{\max}$ is the maximum value of ΔS_M, and $\delta T_{\rm FWHM}$ is a full width at half maximum so-called working temperature span.
